# Simultaneous traumatic dislocation of the posterior tibial tendon and long peroneal tendon: a case report

**DOI:** 10.1186/s13256-021-03036-5

**Published:** 2021-12-13

**Authors:** Johannes Riecke, Max Müller, Andreas Bölderl, Konstantin Genelin

**Affiliations:** 1 Department of Orthopaedics and Traumatology, Medical University of Innsbruck, Tirol Kliniken GmbH, A.ö. Landeskrankenhaus, Universitätskliniken Innsbruck, Anichstraße 35, 6020 Innsbruck, Austria; 2 Present Address: Department of Trauma, Hand and Reconstructive Surgery, Saarland University Hospital, Homburg, Germany

**Keywords:** Posterior tibial tendon dislocation, Peroneal tendon dislocation, Fleck sign, Dislocation

## Abstract

**Background:**

Viewing the existing literature, one can find several documents about dislocation of the peroneal tendons. Clinical findings, diagnostics, and therapy are well described. Instead, the list of documents describing dislocations of the posterior tibial tendon is short. We found no case in which a dislocation of both long peroneal tendon and posterior tibial tendon is described.

**Case presentation:**

We present a case of a 29-year-old male patient who sustained an ankle injury after a fall at a boulder gym. He admitted himself with severe pain, tenderness, and swelling of his left ankle. Dislocation of the posterior tibial tendon and simultaneous dislocation of the long peroneal tendon was diagnosed using x-ray, computed tomography, and magnetic resonance imaging. Transosseous suture repair with periosteal augmentation of the flexor retinaculum was performed at the medial malleolus. At the lateral malleolus, transosseous suture was used to repair the superior retinaculum. The ankle was immobilized following surgery. The patient underwent physical therapy afterwards. The treatment resulted in good recovery, and the patient returned to the same level of performance at rock climbing.

**Conclusion:**

Our novel finding is that simultaneously sustained dislocations of the posterior tibial tendon and the long peroneal tendon may occur and can be successfully treated as if each injury is treated individually. *Level of evidence* Level V, case report.

## Introduction

Posterior tibial tendon dislocation and long peroneal tendon dislocation by themselves are rare traumatic injuries [1–3]. Nonoperative treatment of long peroneal tendon dislocation bears the risk of a high rate of redislocations [4, 5]. Repair of the superior retinaculum in peroneal tendon dislocations with or without fibular groove deepening leads to good clinical results [4]. In case of posterior tibial tendon dislocations, surgery consisting of fixation of the flexor retinaculum or using a periosteal flap technique for tendon sheath stabilization is recommended [5].

To our knowledge, no case of a dislocated peroneal tendon with a simultaneously dislocated posterior tibial tendon has been published in contemporary literature. We present such an injury sustained due to hyperextension in the ankle joint following a fall and the treatment the patient received at our institution.

## Case report

A 29-year-old male patient injured his left ankle after a 2-m fall while bouldering in a climbing gym. During the fall, his left foot caught on a climbing hold, resulting in hyperextension of his ankle joint. He presented himself in the university hospital 2 days after the trauma because of persistent pain and loss of full weight-bearing ability. Severe tenderness at the medial and lateral malleolus was noted during palpation of the ankle joint. Neurovascular function of the foot and ankle was normal, and the skin was uninjured. The patient had never injured his ankles before, and he had not suffered any chronic disease or instability before.

**Fig. 1:**
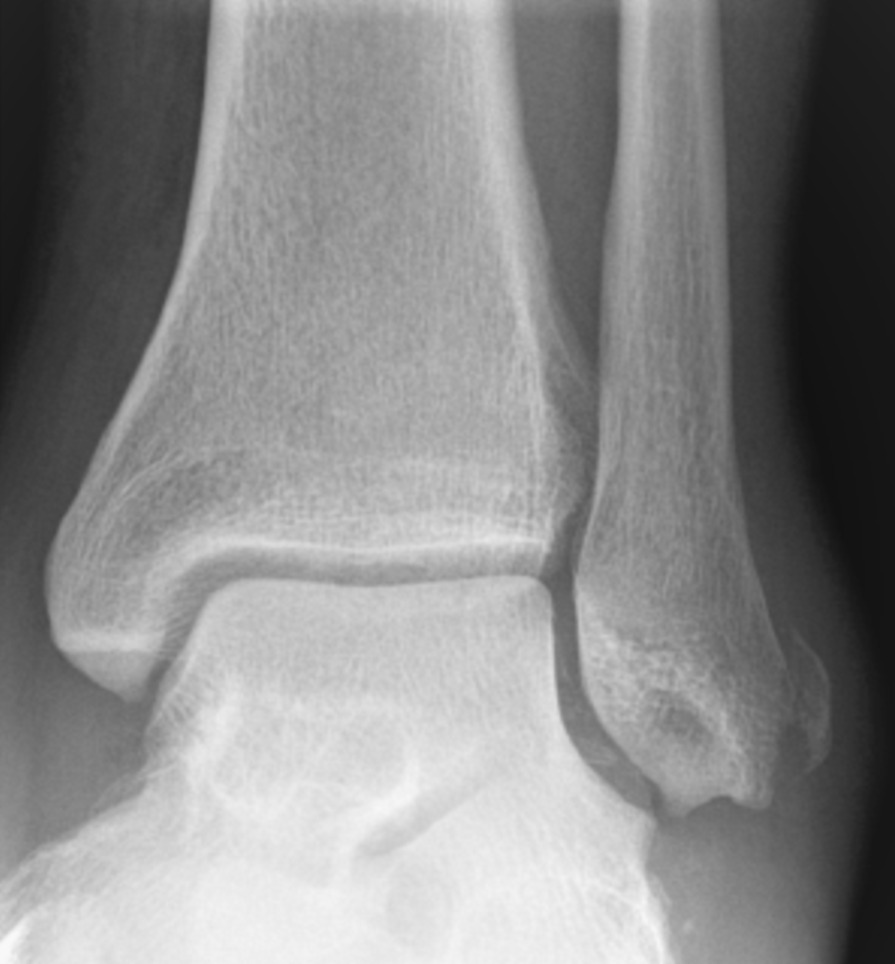
Mortise view x-ray showing a pathognomonic bony avulsion next to the lateral malleolus, the pathognomonic “fleck-sign”

**Fig. 2:**
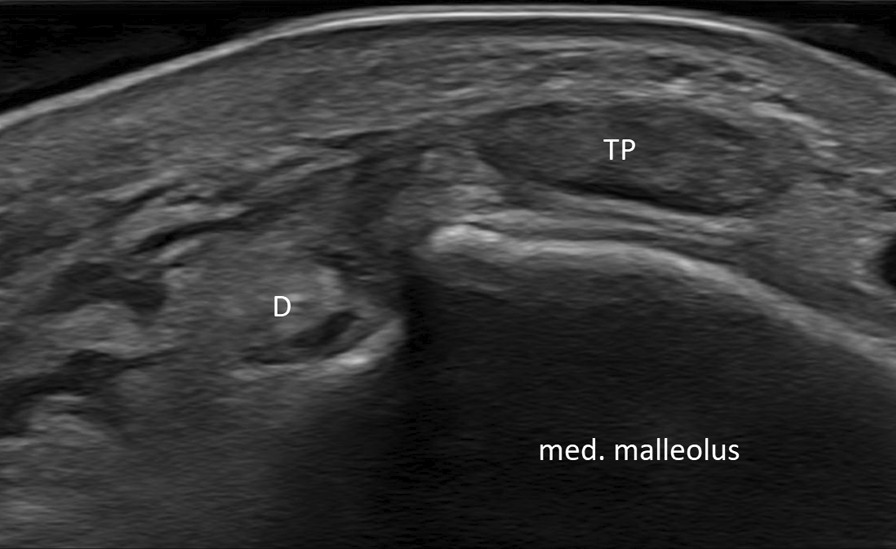
Ultrasound of the left ankle showing the posterior tibial tendon (TP) on top of the medial malleolus. *D* flexor digitorum tendon

**Fig. 3:**
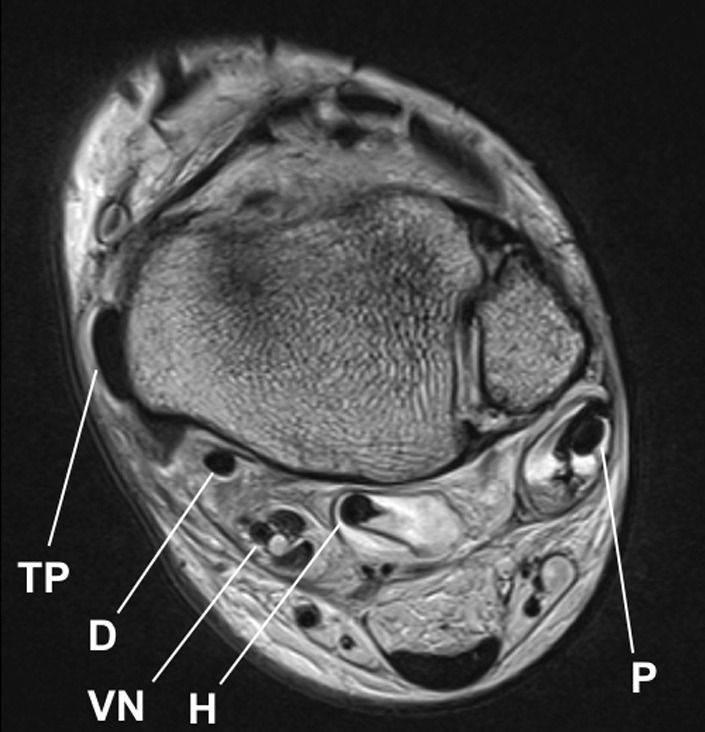
MRI showing the posterior tibial tendon on top of the medial malleolus. *TP* tibial posterior tendon, *D* flexor digitorum tendon, *VN* neurovascular bundle, *H* flexor hallucis longus tendon, *P* long peroneal tendon

Clinical examination showed the posterior tibial tendon palpable mobile on top of the medial malleolus. Specific tests for tendon instabilities such as provocation test according to Orthner and manual dislocation tests [6, 7] could not be performed due to severe pain. X-rays of the ankle as well as computed tomography showed a bony avulsion of the superior retinaculum at the lateral malleolus (Fig. 1). This has been described as “fleck sign” and is highly indicative for peroneal tendon dislocation [8]. No further fractures were found. The peroneal groove at the lateral malleolus was concave. Therefore, no bony correction, such as groove deepening, was planned. Ultrasound and magnetic resonance imaging (MRI) showed the posterior tibial tendon still being dislocated anteriorly (Figs. 2, 3). The long peroneal tendon was no longer dislocated and positioned anatomically behind the lateral malleolus. No tendon lesions were found. An avulsion of the anterior talofibular ligament and a partial lesion of anterior deltoid ligament fibers were found as concomitant injuries. The syndesmotic ligament showed minor sprain but no rupture.

Surgery was postponed until soft-tissue swelling had subsided and a “wrinkle sign” was present 17 days after trauma. Clinical examination showed that the passive sagittal motion was normal, and the hindfoot motion was restricted in inversion and eversion. Active motion was unpleasant due to pain.

The preoperative American Orthopedic Foot and Ankle Society (AOFAS) Score “Ankle Hindfoot Scale” after injury showed a value of 21 out of 100 points (0 pain points, 11 function points, and 10 alignment points).

An 8 cm curved skin incision alongside the posterior tibial tendon was performed behind the medial malleolus.

The flexor retinaculum was found to be ruptured and deficient.

**Fig. 4:**
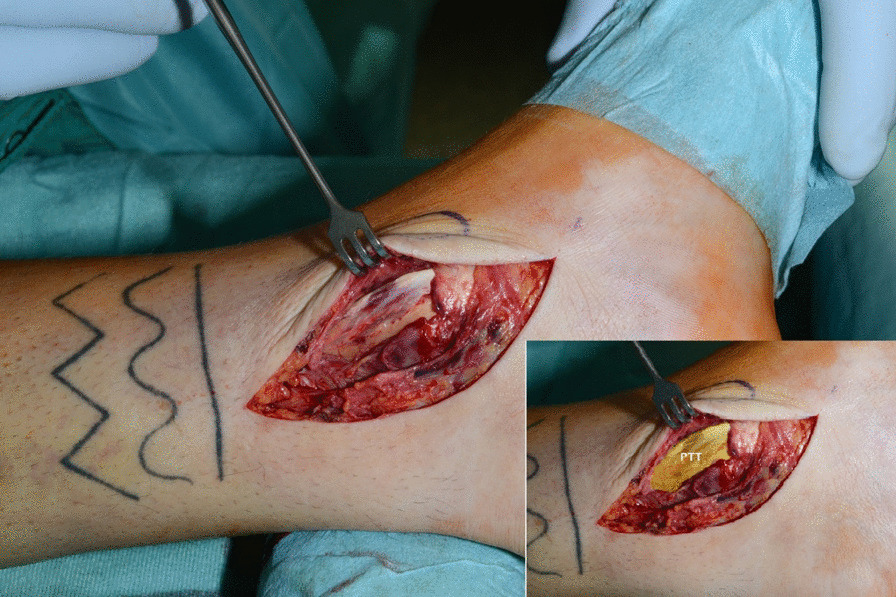
The posterior tibial tendon (PTT, marked yellow) found on top of the medial malleolus

**Fig. 5:**
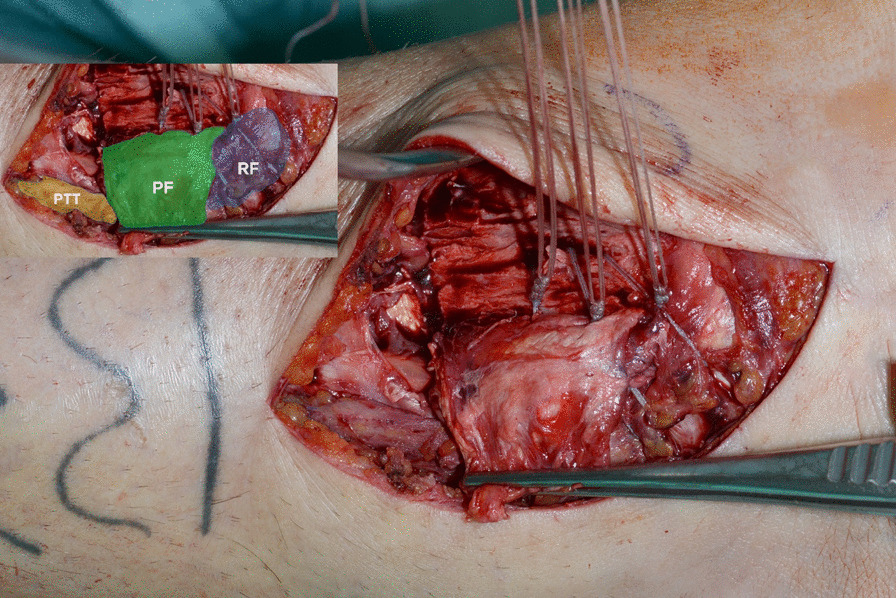
The posterior tibial tendon is seen on the left (PTT, marked yellow), the switched periosteal flap is marked in green in the middle (PF). Distally, the flexor retinaculum (RF, marked blue) is shown already sutured back to the bone entrapping the PTT

The posterior tibial tendon was dislocated anteromedially and found on top of the medial malleolus owing to a posterior tibial tendon dislocation Type 3, according to Strydom (Table 2) [9] (Fig. 4). The tendon was repositioned to its anatomical position. The remaining retinacular fibers were reattached to the posterior aspect of the medial malleolus via two drill holes using a nonabsorbable thread. A periosteal flap augmentation on top of the retinacular flap in vest-over-pants technique completed the procedure (Fig. 5).

An 8 cm posterolateral incision alongside the peroneal tendons was made behind the lateral malleolus. The bony avulsion of the superior retinaculum was verified. The peroneal tendons were found behind the lateral malleolus, but the long peroneal tendon was unstable and could be dislocated with gentle pressure. The injury was classified as an Eckert and Davis Grade 3 [10]. The bony rim and the superior peroneal retinaculum were sutured to the bone through drill holes in the posterolateral aspect of the fibula. Thereby the tendon was stabilized in its anatomic position.

A below-knee cast was applied for 8 weeks postoperatively.

The patient suffered from a punctiform wound healing disorder of 3 × 5 mm size at the distal part of the lateral approach, which was successfully treated with oral antibiotics and needed no operative revision. No further complications occurred.

Initially, no weight-bearing was allowed for the first 6 weeks after surgery. At week 7, full weight-bearing was allowed in the cast. After cast removal, the patient underwent intensive physical rehabilitation. At 3-month follow-up, the patient showed no gait abnormalities. Minor restrictions in range of motion could be observed during physical examination. He was able to follow daily activities and reported no pain. He started to do light sports activity and training. Six months after surgery, the patient reached his preinjury level in climbing and other sports without any noticeable pain and mild restriction due to flexibility while bouldering. He showed no gait abnormality. On clinical examination, we found moderate limitation of sagittal and hindfoot motion. The stability, both subjective and objective, was good. AOFAS-Score “ankle and hindfoot scale” showed a value of 90 out of 100 points (40 pain points, 40 function points, and 10 alignment points).

Although we found mild limitations in mobility on clinical examination, based on patient satisfaction, we decided to adopt a wait-and-see procedure.

**Fig. 6:**
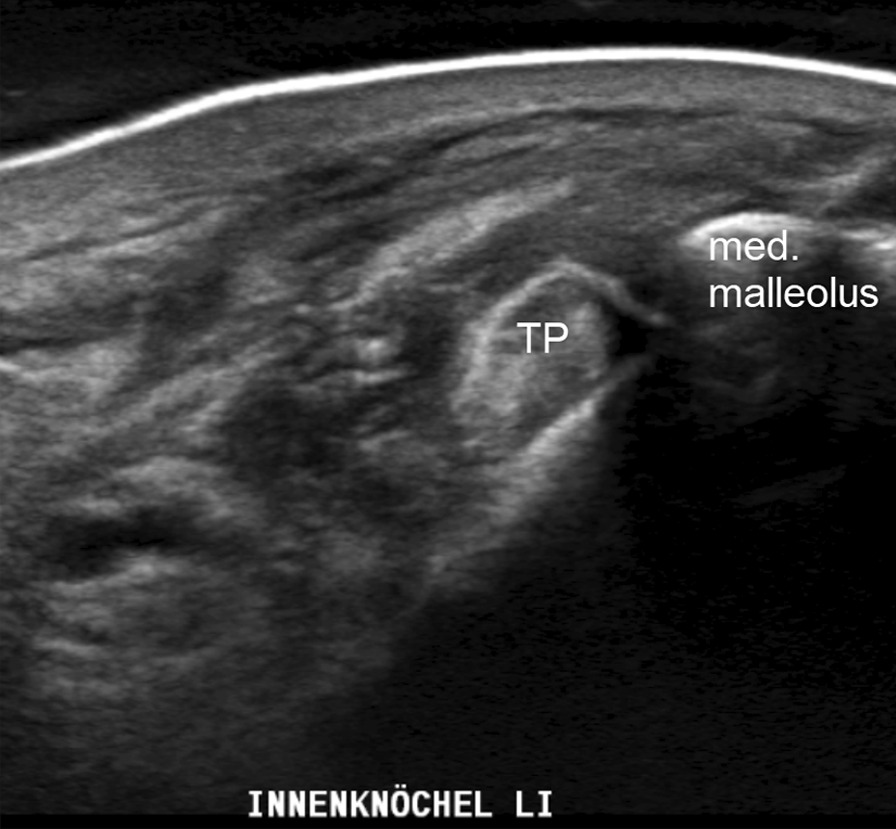
The posterior tibial tendon is seen on the left (TP) behind the medial malleolus

Ultrasound investigation showed the posterior tibial tendon as well as the long peroneal tendon in their former position (Fig. 6).

Until 1 year follow-up, the patient showed no redislocation of both tendons. He had no pain but reported slight limitation of sagittal movement that was not disturbing in any recreational or all-day activities. AOFAS-Score “ankle and hindfoot scale” showed a value of 96 out of 100 points (40 pain points, 46 function points, and 10 alignment points).

## Discussion

Dislocations of the peroneal tendons as well as the tibialis posterior tendons are both rare traumatic injuries. They are often overseen and misdiagnosed as an ankle sprain [2, 11].

Eckert and Davis describe three grades of injury in peroneal tendon dislocation presented in Table 1. Major symptoms are pain and tenderness behind the lateral malleolus. The long peroneal tendon can easily get dislocated by a stress test, performed by elevation of the lateral foot in a squatting position [6, 7]. In grade 3 injuries, the x-ray shows a bony avulsion, the pathognomonic “fleck sign.” According to Toussaint, the “fleck sign” is evidence of the presence of a peroneal tendon dislocation with a specificity of 98% [12]. Grade 1 and 2 injuries are often not visible on native radiological imaging.

**Table 1:** Eckert and Davis Classification for peroneal tendon dislocation [10, 14]

Grade 1	The retinaculum is elevated from the lateral malleolus
Grade 2	The retinaculum is elevated from the lateral malleolus, and the cartilaginous ridge is held on the retinaculum, ruptured from the fibula
Grade 3	The retinaculum is elevated from the lateral malleolus, and the cartilaginous ridge and a cortical rim of bone is held on the retinaculum. A thin bony avulsion can be seen in grade 3 injuries on x-ray as the pathognomonic fleck sign

Even though randomized trials are missing, surgical repair of the retinaculum with or without deepening of the fibular groove leads to good clinical outcome. Oesman depicts it as “a logical, anatomic […] technique” [13]. In the most recent literature, Bakker *et al.* elaborated the plaster cast for 6 weeks as an alternative to operative treatment, but it still correlates with a high rate of failure [11]. On the other hand, van Dijk and colleagues also determined that an operative treatment leads to good clinical results and high satisfaction of the patients [4].

Strydom suggested a classification for posterior tibial tendon dislocations in 2017, presented in Table 2.

**Table 2:** Strydom classification and suggested treatment for posterior tibial tendon dislocation [9]

Type 1—retinacular avulsion (most common)	(a) Retinaculum is avulsed of medial malleolus (b) Retinaculum is avulsed of medial malleolus with a bony rim	(a) Transosseous suture repair (b) Transosseous suture repair or Nonoperative treatment
Type 2—retinacular tear	The retinaculum is ruptured	Repair with sutures
Type 3—deficient tissue	The tissue of the retinaculum is deficient	Reconstruction of the retinaculum with a periosteal flap

The leading symptom is medial ankle pain with tenderness behind the medial malleolus. Persistent swelling and events of snapping and clicking are described as well as sensations of instability and weakness of supination [9]. Due to swelling and pain, a clinical examination is often not possible. MRI can lead to diagnosis in these cases. It can also show concomitant injuries. A dynamic ultrasound investigation can demonstrate dislocations during provocation.

In current literature, operative treatment in tibialis posterior tendon dislocations is recommended predominantly [5]. In the case of an anatomic reposition of the tibial tendon, early diagnosis and consecutive early immobilization and nonoperative treatment can be successful [2].

Strydom classification supposes a treatment for each type (Table 2) [9]: Nonoperative treatment is considered only in type 1b, even though sufficient evidence is missing. The group further suggests surgical repair of the retinaculum (type 2) or reattachment with transosseous sutures (type 1a). In the case of deficient tissue, a periosteal flap technique is suggested (type 3). They recommend to perform a groove deepening in addition to the executed procedure, if the posterior tibial groove is shallow [9].

## Conclusion

Simultaneous dislocations of both the long peroneal and posterior tibial tendons are possible in cases of intensive and powerful dorsiflexion of the ankle.

In cases of discrepancies of clinical findings and severity of the trauma, further thorough examination and diagnostic steps such as CT scan, ultrasound, or MRI are indicated.

Even though there was no previous report of such a case, we were able to show that a combination of established treatment concepts for each injury can lead to a good result.

## Data Availability

Data sharing is not applicable to this article as no datasets were generated or analyzed during the current study.
